# Therapeutic and Diagnostic Antibodies to CD146: Thirty Years of Research on Its Potential for Detection and Treatment of Tumors

**DOI:** 10.3390/antib6040017

**Published:** 2017-11-05

**Authors:** Jimmy Stalin, Marie Nollet, Françoise Dignat-George, Nathalie Bardin, Marcel Blot-Chabaud

**Affiliations:** Inserm UMR-S 1076, Aix-Marseille Université, UFR de Pharmacie, 27 Bd J. Moulin, 13005 Marseille, France; marie.nollet@etu.univ-amu.fr (M.N.); francoise.dignat-george@univ-amu.fr (F.D.-G.); nathalie.bardin@univ-amu.fr (N.B.); marcel.blot-chabaud@laposte.net (M.B.-C.)

**Keywords:** cancer, CD146, antibody, therapy, diagnosis

## Abstract

CD146 (MCAM, MUC18, S-Endo1) is a transmembrane glycoprotein belonging to both CAM and mucin families. It exists as different splice variants and is cleaved from the membrane by metalloproteases to generate a soluble form. CD146 is expressed by numerous cancer cells as well as being one of the numerous proteins expressed by the vascular endothelium. It has also been identified on smooth muscle cells, pericytes, and some immune cells. This protein was initially described as an actor involved in tumor growth and metastatic dissemination processes. Some recent works highlighted the role of CD146 in angiogenesis. Interestingly, this knowledge allowed the development of therapeutic and diagnostic tools specifically targeting the different CD146 variants. The first anti-CD146 antibody designed to study the function of this molecule, MUC18, was described by the Pr. J.P. Jonhson in 1987. In this review, we will discuss the 30 following years of research focused on the detection, study, and blocking of this protein in physiological and pathological processes.

## 1. Introduction

**Figure 1:**
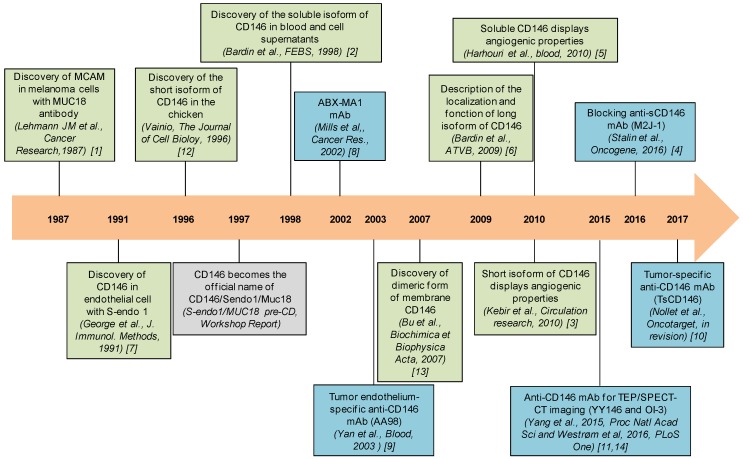
Timeline of research on CD146. This timeline represents the principal discoveries (in green) and antibodies (in blue) related to CD146 [1,2,3,4,5,6,7,8,9,10,11,12,13,14].

Cluster of differentiation 146 (CD146) also named melanoma cell associated molecule (MCAM)/Mucin 18 (MUC18)/S-Endo 1 was first discovered in metastatic melanoma and its expression was rapidly associated to a bad prognosis. Since this initial discovery, CD146 was found to be upregulated in many cancer types. Thereafter, CD146 was also identified on endothelial cells where it is involved in angiogenesis. Angiogenesis, which corresponds to the formation of new blood vessels from pre-existing vessels, largely contributes to tumor development. The identification of new proteins and cells involved in the development and dissemination of tumors remains an important challenge to generate diagnostic and therapeutic tools. For several decades, studies have highlighted CD146 as a key actor of tumor growth and angiogenesis. CD146 displays different isoforms and could thus constitute a novel therapeutic target. Many studies describe structural features, localization, and functions of CD146 in the vascular endothelium and tumor cells. The timeline of research on CD146 is presented in Figure 1.

This review will now focus on the role of the different isoforms of CD146 with a particular interest in the diagnostic and therapeutic tools targeting the molecule.

## 2. Description and Cellular Localization of CD146

### 2.1. Genomic Description of CD146

The CD146 gene is located specifically on the arm q23.3 of chromosome 11 in humans and on chromosome 9 in mice. The gene encoding the CD146 protein extends over 14kb. CD146 structure is composed of five immunoglobulin-like domains, two variable domains of V2 type, and three constant domains of C2 type, but also a transmembrane domain and an intracytoplasmic portion [15]. The extracellular part of the molecule, including the five immunoglobulin domains, is encoded by 13 exons. The transmembrane and intracellular domains are encoded by three exons.

The promoter of CD146 presents different putative binding sites and motifs including AP1, AP-2, cAMP Response Element (CRE), SP1, CArG, and c-myb. Analysis of this DNA segments suggests that the four SP-1 sites, the two AP-2 domains, and one response element to Adenosine MonoPhosphate cyclic (AMPc)-(CRE) form the minimal promotor of CD146 [16]. Specific sites play a role in CD146 expression. The AP-2 sites which are located at −131 and −302 by relative to the initial ATG, reduced promoter activity by 70% and 44%, respectively [16]. Moreover, when mutated, the CRE site inhibits by 70% the transcription of the gene. Therefore, AP-2 [17] and CRE sites [18] have been described to modulate CD146 expression in melanoma cells, leading to an increase in tumor growth and metastatic potential in these cancers. In fact, the AP-2 binding site located in the promoter (located at −23 bp) is an inhibitor of the transcription of CD146 while the other AP-2 sites (located respectively at −131, −302, and −505) are transcription activators [16,17].

The CD146 mRNA has been first identified in human melanoma cancer cells and represents 3.3 Kb [19]. Its encoding region is about 1940 bp. There exists a strong homology between the human and mouse mRNA sequences, but several differences can be noted. In humans, there is a lengthening of the 3’ and 5′ Untranslated Transcribed Region (5’ UTR) region as wells as a loss of 6pb in exon 2. The encoding regions and 5′UTR region have a homology of about 80% and 72%, respectively, between the murine and human genes and there is only 31% of homology for the 3′UTR region. Finally, the protein sequence has about 76% homology between these two species [1,20].

There is one polymorphism concerning CD146 which has been described. In fact, rs3923594 polymorphism is associated with stages, metastasis, and recurrence in clear cell renal cell carcinoma in the Chinese population [21].

### 2.2. CD146 Protein Structure and Isoforms

The CD146 protein sequence derived from the coding region has a theoretical molecular weight of about 72 kilodaltons. However, CD146 molecular weight is around 113 kilodaltons. This difference is due to the glycosylation sites which are present on the protein sequence. In fact, glycosylations represent approximately 35% of the total molecular weight of the protein with mostly N-glycosylations. The presence of sialylation has also been shown [22].

As a member of the immunoglobulin superfamily, CD146 displays many similarities in the structure with BCAM (B-cell adhesion molecule) and ALCAM (activated leukocyte cell adhesion molecule), but also many similarities in functions and expression in tumor and endothelial cells [23].

A short isoform and a long isoform have been identified for membrane CD146. These two isoforms are generated by alternative splicing on exon 15 causing a reading frame shift. In 1987, the long isoform was discovered in human melanoma and, more recently, the short isoform was identified as a complementary DNA from chicken [24]. In addition to these two membrane isoforms, a soluble form of CD146 was identified in human endothelial cell culture supernatant (HUVEC) and in the bloodstream of patients [2].

The extracellular portion of both membrane isoforms of CD146 is similar. The difference is located in the intracytoplasmic portion [24]. In fact, the intracytoplasmic domain of the short isoform is shorter and contains a PDZ-binding domain and a phosphorylation site for protein kinase C. The long isoform is composed of an endocytosis signal sequence and two Protein Kinase C (PKC) phosphorylation domains [24].

Both mice and human intracytoplasmic domain sequences are similar at 95% and 93% for the short isoform and long isoform, respectively. This conservation across species is in accordance with the important functions carried by the intracytoplasmic domain of CD146.

Finally, identified for the first time in 1998 [2], a soluble form of CD146 with a molecular mass around 100 Kilodaltons was detected in human plasma [25], resulting from a metalloprotease-dependent shedding of the extracellular domain [26].

### 2.3. Cellular Localization of CD146

In CD146-positive cancer cells, both isoforms of CD146 are expressed but their precise localization remains to be defined.

CD146 localization was essentially studied in endothelial cells. The localization of short and long membrane CD146 isoforms is different. The long isoform of CD146 is expressed at the intercellular junctions. Co-staining between CD146 and VE-cadherin, Focal Adhesion Kinase (FAK), Platelet Endothelial Cell Adhesion Molecule (PECAM), and the catenin/cadherin complex shows no co-localization, suggesting that the long isoform of CD146 is not located in the adherent junctions, tight junctions, or focal adhesion sites [27,28]. An immunohistochemical staining of this isoform in endothelial colony forming cells (ECFC) confirmed the junctional localization of this protein. Moreover, the presence of a cytoplasmic pool of long CD146 that can be redistributed to the cell membrane was also evidenced [3]. Overexpression of the long isoform of CD146 in MDCK cell line (Madin–Darby canine kidney) showed a baso-lateral localization of the protein and this localization was dependent on the presence of a di-leucine motif on its intracytoplasmic peptide sequence [27]. 

In contrast, short CD146 shows an apical localization in ECFC [3]. An apical localization was also evidenced in MDCK cells [27]. Short CD146 isoform is involved during the first step of angiogenesis process. It is involved in the adhesion, migration, and proliferation of endothelial cells [3].

The confluence of the endothelial monolayer constitutes a regulator of the cellular distribution of the two membrane isoforms. Indeed, in non-confluent endothelial cells, the long isoform was localized in the cytoplasm and was not detected at the cell membrane. Concerning the short CD146 isoform, it was localized in the nucleus and at the migration front in non-confluent cells [3]. In other experiments performed on chickens, results showed that the long isoform of CD146 was preferentially localized in the microvilli where the protein plays a role in their formation [29].

## 3. CD146 in Cancer Cells

CD146 has been identified for the first time in melanoma where it plays an important role in the disease progression. Thereafter, CD146 has been shown to be expressed in various cancers, such as pancreatic, breast, prostate, ovarian, lung, kidney cancers, osteosarcoma, Kaposi sarcoma, angiosarcoma, Schwann cell tumors, children and adult acute B cell lymphoblastic leukemia, or leiomyosarcoma [30]. The mechanism of this neo-expression is still largely unknown but, in prostate cancer, it was reported that high expression of CD146 resulted from hypermethylation at the promoter of the CD146 gene [31]. A strong correlation was found between the increased metastatic capacities of tumor cells and the increased expression of this molecule [32]. CD146 expression in human melanoma cell lines correlates strongly with their ability to form primary tumors and to generate distant metastasis in both immunodeficient nude and Severe Combined ImmunoDeficiency (SCID) [33]. As an example, CD146 expression increased melanoma cell metastasis in lung in nude mice after intravenous injection [34]. These results were confirmed by using interfering RNA directed against CD146 which decreased migration, proliferation, and invasion of melanoma cells in vitro [35]. Different cancer markers were analyzed in the early and late stages of patients with melanoma. CD146 was the only protein in blood sample analysis which correlated with the advanced stages of the cancer [36]. This finding was confirmed in another study where CD146 constituted a marker of poor prognosis and survival in melanoma patient. It was concluded that CD146 was a better marker of disease progression than analysis of sentinel lymph node on biopsies [37].

Likewise, in prostate cancer, CD146 overexpression increased tumor growth, invasiveness, and metastatic potential [38]. This result was confirmed in biopsies of patients where CD146 overexpression was also observed. In this study, CD146 expression was correlated with a poor prognosis. CD146 expression was also correlated with an increase in the metastatic potential in ovarian carcinoma. Furthermore, inhibition of CD146 protein expression in ovarian cancer cell lines inhibited invasiveness and tumor spread, and induced apoptosis. This was explained by the fact that a lack of CD146 induced a decreased activity of Rho GTPase [39] which is involved in these processes.

In breast cancer, it was first reported that CD146 could act as a tumor suppressor [40] while more recent studies have described CD146 as a poor prognosis marker [41]. Indeed, over-expression of CD146 in a breast cancer cell line increased both motility and tumorigenicity [42]. This result was confirmed in vivo.CD146 expression was correlated with markers of EMT in both gastric and breast cancer patient biopsies [43,44]. This is due to the fact that CD146 induced the epithelial–mesenchymal transition (EMT) in gastric and breast cancer cells in vitro. In triple negative breast cancers, the increased expression of CD146 in epithelial cells induced a loss of epithelial markers and an increase in mesenchymal markers expression, leading to increased invasiveness, migration, and mammospheres formation. In this study, it was shown that CD146 induced an increased expression of CD44 but also a decreased expression of CD24 on the cell membrane suggesting that cancer cells acquire a cancer stem cell-like phenotype [45].

It is important to note that all the mechanisms involved in melanoma progression are not still understood. A recent study showed that a B lymphocyte subpopulation, called B1 lymphocytes, displayed a pro-metastatic potential. In fact, a decrease of both tumor growth and metastatic dissemination was observed after depletion of this cellular population in an experimental metastatic model. The homophilic interaction between the B lymphocytes and the cancer cells, through CD146, was responsible for the metastatic dissemination [46]. Moreover, the expression of CD146 at the cell membrane of cancer cells was increased when these cells were co-cultured with the B1 lymphocytes, leading to an increase in the number of metastasis.

A recent study reported that cancer cells expressing CD146 were able to secrete soluble CD146 in vitro and in vivo [4]. This phenomenon was described in melanoma, colorectal cancer, and pancreatic cancer cell lines by Enzyme-Linked ImmunoSorbent Assay (ELISA). The authors demonstrated that soluble CD146 secreted by cancer cells induced both autocrine effects on cancer cells and paracrine effects on vascular endothelial cells. Furthermore, cancer cells stimulated in vitro with recombinant soluble CD146 were more proliferative and produced more pro-tumorigenic and angiogenic factors, such as Vascular Endothelial Growth Factor (VEGF). The authors demonstrated that soluble CD146 induced an anti-apoptotic phenotype in cancer cells and that it could also decrease cancer cell senescence visualized by beta-galactosidase staining. The c-myc signaling pathway was also upregulated by soluble CD146 confirmed by an increased protein expression of c-myc, phospo-c-myc on residue ser62 and max, a partner of c-myc. Finally, they also showed that soluble CD146 secreted by cancer cells increased endothelial cell proliferation and tumor angiogenesis. These pro-angiogenic and anti-apoptotic effects were confirmed in vivo in different models of xenografted mice. 

In line with this result, an increased concentration of soluble CD146 in blood of patients with non-small cell lung cancer has been described, as compared to control patients or patients with respiratory inflammatory disease [47]. Altogether, these results show that soluble CD146 also plays a major role in tumor development and angiogenesis.

## 4. CD146 in Angiogenesis; Immunity and Inflammation

Initiation and progression of cancer are closely linked to angiogenesis, immunity, and inflammation. Of interest, CD146 is expressed in many cells involved in these different processes.

Angiogenesis plays a major role in tumor development. Endothelial cells which display angiogenic properties can proliferate, migrate, adhere, and generate new capillaries from pre-existing vessels. The discovery of the existence of the two membrane isoforms of CD146 and the description of a soluble form of CD146 led to study their implications during angiogenesis. Using specific siRNA directed against the two membrane isoforms, it has been shown that the absence of short CD146 in endothelial cells decreased cellular proliferation, migration, and adhesion. Conversely, its overexpression displayed opposite effects. In contrast, the absence of long CD146 isoform destabilized the junctions of neo-vessels which were necessary to generate pseudo-capillaries in matrigel in vitro. So, both short and long CD146 isoforms display complementary effects for the generation of neo-vessels. The pro-angiogenic effect of short CD146 was confirmed in vivo by transplantation of ECFC over-expressing this protein in a mouse model of hind limb ischemia. An increase in both ECFC incorporation in ischemic muscle and neo-vessel generation was observed [3]. Recent works showed that the short isoform of CD146 was associated with different proteins at the membrane of endothelial cells including VEGF-R2 [48], angiomotin, and VEGF-R1 [48,49,50]. This association seems to be essential for the activation and function of these different pathways. Indeed, the absence of short CD146 isoform decreased both phosphorylation of VEGF-R2 and pro-angiogenic effects of VEGF in endothelial cells. It was also recently shown that Galectin 3 constituted a new partner for CD146 in endothelial cells and that this association induced a dimerization of CD146 at the cell surface, leading to the activation of several pathways. Among them, the AKT pathway leads to the secretion of metastasis-promoting cytokines [51]. Finally, it has also been shown that soluble CD146 displayed a pro-angiogenic activity. Soluble CD146 was able to increase the formation of pseudo-capillaries in vitro but also to induce neo-vascularization in a rat model of hindlimb ischemia. Moreover, recruitment of mature and immature endothelial cells but also smooth muscle cells was increased in matrigel plugs containing soluble CD146 in mice, leading to the formation of capillary-like structures [5]. Interestingly, it was shown that soluble CD146 stimulated short CD146 isoform through its binding on angiomotin [49] and that the angiogenic properties of soluble CD146 were additive to those of VEGF [5].

CD146 is also expressed in approximately 1% of blood mononuclear cells of peripheral blood in healthy patients. More precisely, a flow cytometry analysis of the different lymphocyte populations showed that CD146 was expressed on a sub-population of B and T human lymphocytes [52]. About 1% of B lymphocytes express CD146 and its expression is upregulated by different cytokines such as IL-4 and CD40. In addition, CD146 can be neo-expressed on some immune cell populations after stimulation [52]. These results have been deepened by using two antibodies generated in rat. Using the HUT102 T lymphocytic cell line, CD146 was shown to be expressed on 2% of CD3+, CD3+/CD4+, and CD3+/CD8+ lymphocytes. Here again, stimulations with cytokines such as IL-2 [52] and PHA (phytohemagglutinin) [53] increased the amount of CD146+ T lymphocytes. These cells were also found in vivo in the synovial fluid of patients with rheumatoid arthritis [53]. In mouse, a leucocyte screening was carried out and demonstrated that both T/B lymphocytes, monocytes, and dendritic cells did not express CD146. Moreover, it was also demonstrated that CD146 was expressed by 30% of neutrophils and 60% of Natural Killer (NK) cells. Furthermore, there was a correlation between expression of CD146 and an increased expression of CD11b and CD27 which reflected the NK maturity. The CD146 positive NK produced gamma interferon in smaller quantities and also had a decreased cytotoxic capacity [54].

Different studies have reported that CD146 is also involved in inflammation, and in particular in leukocyte transmigration [6]. Using both a monocyte cell line, THP-1, and freshly isolated monocytes, it was shown that CD146 modulated monocyte transmigration. Indeed, junctional CD146 expressed by endothelial cells bound to monocytes through a heterophillic interaction in order to increase their transmigration. Furthermore, monocyte transmigration was increased after a stimulation with soluble CD146 [6]. In addition, an increased adhesion capacity of CD146+ T lymphocytes was observed after stimulation with IL-1 beta. This effect was blocked by adding an anti-CD146 antibody [55]. An inflammatory stimulus could also increase the adhesion of CD4+/CD146+ T lymphocytes on endothelium. In this study, transfection of the long isoform of CD146 in NKL.1 cell line reduced the number of rolling cells and increased their adhesion to endothelial monolayer. Furthermore, an increase in microvillis in T lymphocyte cell membrane was observed. An increased transmigration of lymphocytes through HMVECs (human microvascular endothelial cells) monolayer following incubation with an anti-CD146 antibody (P1H12) was reported in another study [56]. Finally, a co-expression of CD146 and CCR6 was demonstrated in a population of TH17 lymphocytic cells [57].

## 5. Approaches for Therapeutic Targeting of CD146

In view of the major role of CD146 in tumor pathologies, different antibodies have been generated. These different antibodies, their utilization and their generation are summarized in Table 1.

In 1987, Pr. J.P. Jonhson identified CD146 as a marker of melanoma progression by using an antibody (called MUC18) generated by mouse immunization with a cell lysate of metastazing melanoma [58].

Later, the team of Pr. F. Dignat-George identified CD146 as a new marker of circulating endothelial cells in the blood by flow cytometry. This result was obtained by the generation of a mouse monoclonal antibody named Sendo-1 [7] obtained by mice immunization with a HUVEC lysate [59,60].

Since these pioneer antibodies, different antibodies directed against CD146 have been generated in order to block its functions. The first one, named ABX -MA1, was able to recognize the human form of CD146. It could inhibit the formation of spheroids containing melanoma cells and could reduce metastasis, tumorigenicity, and vascularization of tumors in vivo. These effects were due to the inhibition of MMP-2 expression and activity that was involved in metastasis dissemination [8]. In another model of immunodeficient mouse, injection of ABX-MA1 significantly decreased metastatic dissemination of cells derived from human osteosarcoma in lung [61].

Another strategy was developed to target tumor stromal cells. To this end, monoclonal antibodies specifically directed against the vascular endothelium of tumors were generated. During the antibody screening, the authors focused on one antibody named AA98. AA98 mAb recognized CD146 expressed on the intra-tumoral vasculature but not on the vasculature of healthy tissues [9]. This antibody recognized CD146 and inhibited its activity. Indeed, the injection of AA98 mAb decreased the number by 70% the number of vessels in a chicken chorrioallantoic membrane assay. Moreover, AA98 antibody reduced tumor angiogenesis in xenografted tumors (hepatocellular carcinoma, pancreatic, and leiomyosarcoma) in mice [36]. Following this first work, authors investigated the signaling pathways altered by this antibody. They demonstrated that it modulated phosphorylation of several kinases such as p38/MAPK, suppressed NFkB activation, and inhibited both MMP-9 and ICAM-1 expression. These results suggested that inhibition of NFkB activity was a major point of the inhibitory effects of AA98 and explained the decrease in cell migration, angiogenesis, and metastatic dissemination [62].

It is worth noting that AA98 mAb displayed additive inhibitory effects when used in combination with bevacizumab (anti-VEGF antibody) in xenografted models of both human pancreatic and melanoma tumors [64]. It also displayed synergistic inhibitory effects when associated with vorinostat for the treatment of ovarian cancer [65]. Finally, this antibody significantly reduced colonic chronic inflammation in a mouse model and prevented the development of cancer associated with the inflammatory state [48]. Several antibodies were generated but they did not display therapeutic effects (see Table 1).

Recently, a novel inhibitory antibody against the soluble form of CD146 was generated. This antibody named M2J-1 is specific of the soluble form of CD146 [4]. The authors demonstrated that M2J-1 antibody treatment was able to decrease tumor growth and angiogenesis in human melanoma and pancreatic tumors xenografted in immunodeficient mice. It could also induce tumor apoptosis in vivo. The particularity of this antibody is that it does not bind to membrane CD146, limiting the side effects of antibodies which also target CD146 expressed at the surface of the vascular endothelium.

Recently, a novel antibody specifically recognizing the tumor form of CD146 was generated. This antibody, referred to as TsCD146 mAb, could detect CD146 in cancer cells but not in cells that express the molecule in physiological conditions (endothelial cells, smooth muscle cells). TsCD146 mAb detects specifically tumor CD146 in human biopsies and in animal models of xenografted tumor cells by positron emission tomography (PET) imaging. In addition, TsCD146 mAb could specifically detect CD146-positive cancer microparticles in the plasma of patients with melanoma. TsCD146 mAb also displayed therapeutic effects since it could reduce the growth of human CD146-positive cancer cells xenografted in nude mice. This effect was due, at least in part, to a decrease in the proliferation of CD146-positive cancer cells after TsCD146-mediated internalization of the membrane CD146. This antibody could be of major interest for diagnostic and therapeutic strategies against CD146-positive tumors in a context of personalized medicine [10]. 

To date, there is only one humanized anti-CD146 antibody available. It has been developed by Prothena Corporation plc (Nasdaq-PRTA, Dublin, Ireland) and it is named PRX003. It was used in clinical trial for treatment of patients with psoriasis. The pharmacodynamics effects did not translate into meaningful clinical benefit in patients with psoriasis treated with PRX003 (http://ir.prothena.com).

**Figure 2:**
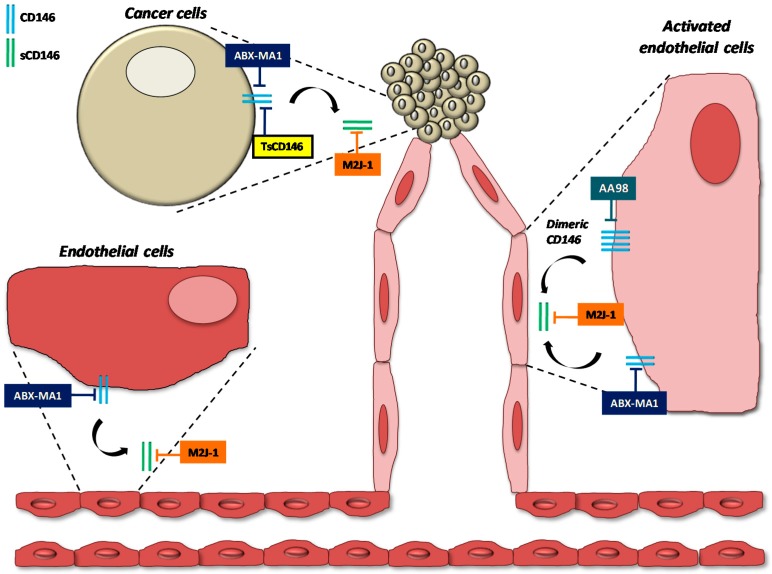
Targets of therapeutic anti-CD146 antibodies. The targets of the different therapeutic anti-CD146 antibodies are indicated on endothelial and cancer cells.

The targets of the different therapeutic anti-CD146 antibodies are indicated in Figure 2.

Furthermore, a meta-analysis with 12 studies involving 2694 participants reveals that the associations between CD146 overexpression and the outcome endpoints (overall survival or time to progression) are statistically significant in Mongoloid and Caucasian patients with both lung and digestive system cancer. Showing as well as CD146 could be a prognosis predictive biomarker [66].

## 6. New Approaches for Theranostic Targeting of CD146

Early detection of tumors constitutes the leading strategy to extend patient survival. Due to the availability of different imaging technics, new concepts emerged for non-invasive diagnosis of cancer using magnetic resonance imaging (MRI), computed tomography (CT), and contrast-enhanced ultrasound (CEUS) [67,68,69]. However, these imaging techniques allowed the detection of tumors that are seldom visible during the first stages of development or tumors which are insensitive to biological changes occurring soon after treatment [67,68,69]. These techniques are more efficient to non-invasively detect both dysregulations of tumor-associated markers and variations in tumor metabolism or in molecular pathways. Novel technologies, such as positron emission tomography (PET) could improve tumor detection, but they need the identification of tumor-specific molecular signatures and antigens. Therefore, the generation of targeted imaging agents with high affinity for the selected molecule constitutes an important step. ImmunoPET exploits the binding affinity and specificity of monoclonal antibodies and peptides for their cognate antigen and binding protein, respectively, to detect tumor-associated proteins. It has been assessed that immunoPET has a better sensitivity and specificity compared to MRI and PET [70].

In this way, CD146 has emerged as an attractive target for targeted diagnosis and therapy in cancer.

In very recent studies, CD146 detection by immunoPET imaging was described for several types of tumors. By using a newly generated monoclonal antibodies named YY146, CD146 was suggested as a new target for the early detection and better staging of hepatocellular carcinoma (HCC). It could also be used for both monitoring of tumor response to CD146-immunotargeted therapies and image-guided ablation of tumors for malignant brain cancer [71,72]. ImmunoPET imaging using YY146 antibody coupled with ^64^Cu-NOTA or ^89^Zr-Df demonstrated the potential use of this radiotracer for imaging lung [73] or brain [72] cancer, respectively, using different human lung cancer cell lines in immunodeficient mice. Moreover, the intensity of labeling was proportional to the intensity of expression of CD146 in these cell lines [73]. Another antibody targeting CD146, called OI-3, labeled with 125-I or 177-Lu was also used to detect CD146 expression in osteosarcoma xenograft implanted subcutaneously in nude mice [11].

## 7. Conclusions

The expression of CD146 is correlated with tumor growth and metastatic dissemination. Moreover, the soluble form of CD146 secreted by both cancer and endothelial cells is involved in tumor growth and angiogenesis. So, CD146 constitutes an important target for the early detection and treatment of CD146-positive tumors in a context of personalized medicine. This is now well understood and many diagnostic and/or therapeutic antibodies to CD146 were generated for this purpose.

## Figures and Tables

**Table 1:** Description of available anti-CD146 antibodies.

Antibody	Utilization	Antibody generation
MUC 18	IHC, WB	Mouse immunization with a cell lysate of metastazing melanoma (Lehmann JM et al., Cancer Research, 1987) [1].
S-endo 1 (Biocytex)	IF, IHC, WB, FC	Mouse immunization with a HUVEC lysate (George et al., J. Immunol. Methods, 1991) [7].
7A4 (Biocytex)	IF, WB, FC, ELISA, IP	Mouse immunization with an injection of CD146
AA98	Bloquing in vivo, WB, IHC, IF, WB	Mouse immunization with a lysate of HUVEC stimulated with conditioned medium of hepatoma cell line SMMC 7721 (Yan et al., Blood, 2003 ) [9].
ABX-MA1	WB, FC, IHC	Generated using Abgenix’s proprietary XenoMouse mice (Mills et al,, Cancer Res., 2002) [8].
M2J-1	Bloquing in vivo, ELISA	Rat immunization with an injection of recombinant soluble CD146 (Stalin et al., Oncogene, 2016) [4].
TsCD146	IF, FC, WB, PET/SPECT-CT, bloquing in vivo	Rat immunization with an injection of recombinant soluble CD146 (Nollet et al., in process) [10].
YY146	PET/SPECT-CT	Mouse immunization with an injection of the human CD146 antigen (Yang et al., 2016, Proc Natl Acad Sci) [14].
OI-3	Radiolabeling, FC	Mouse immunization with an injection of recombinant chimeric versions of human IgG1 or human IgG3 Fc sequences (Westrøm et al, 2016, PLoS One) [11].
5G6 (Biocytex)	IF	Mouse immunization with an injection of immunopurified CD146
2F6 (Biocytex)	IF	Mouse immunization with an injection of immunopurified CD146
3D9 (Biocytex)	IF, FC	Mouse immunization with an injection of immunopurified CD146
Antibody against short or long isoforms of CD146	WB, IF, FC, IP	Rabbit immunization with an injection of peptides corresponding to the intracellular part of short and long CD146 (Kebir et al., Circulation research, 2010) [3].
P1H12 (Abcam)	IF, IHC, WB, FC, IP, ELISA	Mouse immunization with a HUVEC lysate (Solovey et al., J Lab Clin Med, 2001) [56]
EPR3208 (Abcam)	IF, IHC, FC, WB	Rabbit immunization with an injection of a synthetic peptide corresponding to Human CD146 AA 600 to the C-terminus
541-10B2 (Miltenyi Biotec)	FC	Mouse immunization
AA1	WB	Mouse immunization with CD146 purified from HUVEC (Zhang et al., Hybridoma, 2008) [63]

In this table are listed the different anti-CD146 antibodies, their characteristics and how they were generated. IHC = immunohistochemistry, WB = Western blot, IF = immunofluorescence, FC = flow cytometry, IP = immuno-precipitation, PET = positron emission tomography, ELISA = enzyme-linked immunosorbent assay, SPECT-CT = single-photon emission tomography / computed tomography.
